# Maternal anemia at admission for labor in twin pregnancies: an indicator of adverse maternal and neonatal outcome

**DOI:** 10.1007/s00404-026-08352-z

**Published:** 2026-02-09

**Authors:** Tzuria Peled, Yael Levitt, Ariella Tvito, Sorina Grisaru-Granovsky, Misgav Rottenstreich

**Affiliations:** 1 Department of Obstetrics & Gynecology, Shaare Zedek Medical Center, Faculty of Medicine, Hebrew University of Jerusalem, 12 Bayit Street, 91031 Jerusalem, Israel; 2 Faculty of Medicine, Hebrew University of Jerusalem, Jerusalem, Israel

**Keywords:** Anemia, Twins pregnancy, Perinatal outcomes, Neonatal outcomes, Postpartum hemorrhage, NICU

## Abstract

**Purpose:**

Maternal anemia during pregnancy is associated with adverse obstetrical outcomes. This study aimed to assess maternal and neonatal outcomes in women with anemia in twin pregnancies, compared to women with normal hemoglobin levels.

**Methods:**

A multicenter retrospective cohort study was conducted including women with twin pregnancies who delivered at 24–42 weeks between 2005 and 2021. Maternal and neonatal outcomes were compared between those who had diagnosis of maternal anemia upon admission for labor (hemoglobin < 11 g/dL), to those who have normal hemoglobin level. The primary outcome was composite adverse neonatal outcomes. Univariate analysis was followed by multivariate analysis to control potential confounders.

**Results:**

During the study period, there were 5,530 twin deliveries; 5,004 women met the inclusion criteria. The maternal anemia prevalence upon admission was 16.8% (*n* = 840). After controlling for potential confounders, we found an independent association between maternal anemia in twin pregnancies and composite adverse neonatal outcomes for both twins—aOR 1.81 (1.55–2.12) for twin A and aOR 1.77 (1.51–2.06) for twin B. Anemia was also independently associated with higher risk for preterm delivery, cesarean delivery, maternal blood product transfusion and NICU admission for both twins.

**Conclusions:**

Maternal anemia in twin pregnancies is associated with an increased risk of adverse maternal and neonatal outcomes. Clinicians should be aware of this condition, consider appropriate interventions for correcting the anemia, and ensure close monitoring of both the mother and the neonates. Further research is warranted to evaluate the effectiveness of anemia correction strategies in reducing obstetric burden.

## Introduction

Anemia is defined as a hemoglobin (Hgb) level below 11 g/dL [1]. The World Health Organization (WHO) estimates that approximately 40% of pregnant women worldwide are affected by anemia, making it a significant public health concern during pregnancy [2, 3]. Iron deficiency is the most common underlying cause, although anemia might be result from hereditary disorders, folate or vitamin B12 deficiency, chronic disease, hemolysis, or aplastic anemia [1, 3]. Despite its relatively straightforward diagnosis and treatment, anemia in pregnancy is frequently underdiagnosed and inadequately managed by healthcare providers [4].

Maternal anemia has been associated with a range of obstetric complications affecting both the mother and the fetus. Anemic pregnant women are at an increased risk for placental abruption, preterm delivery (PTD) with its attendant complications of prematurity, low neonatal birthweight, stillbirth, and maternal mortality [2, 3, 5–7]. It has also been linked to an increased risk of postpartum hemorrhage (PPH) and the need for blood transfusions [8–10]. 

In addition, prenatal maternal anemia has been linked to long-term implications. It was noted as a risk factor for maternal cardiovascular morbidity [11], as well as association with long-term offspring infectious [12], neurological [13] and respiratory morbidity [14].

The incidence of twin pregnancies escalated markedly in recent decades, accounting for approximately 3.12% of all pregnancies [15]. This is attributed to increased rate of late motherhood and use of assisted reproductive technologies [16]. While many twin pregnancies result in favorable outcomes, they are inherently associated with a higher risk of complications, including PTD, pre-eclampsia, PPH [17], and cesarean delivery [18], which overlap with the known risks associated with anemia. Furthermore, the prevalence of maternal anemia is significantly higher in twin gestations compared to singleton pregnancies [19, 20].

Despite this elevated risk, few studies have specifically investigated the impact of maternal anemia on maternal and neonatal outcomes in twin pregnancies, existing evidence is limited and inconsistent. Some studies have suggested that anemia in twin pregnancies is associated with adverse outcomes, such as low 1-min Apgar scores, reduced neonatal birthweight [21], and increased need for maternal blood transfusions [22]. Correction of anemia in the third trimester has been shown to improve outcomes. [21].

However, other studies have found no significant association between anemia and key outcomes, such as preterm delivery [23].

This study aimed to investigate whether maternal anemia upon admission for labor in twin gestations is associated with an increased risk of adverse neonatal and maternal outcomes.

## Materials and methods

### Study design and population

This multicenter retrospective cohort study was conducted using electronic medical records from two university-affiliated medical centers in Jerusalem, Israel—Shaare Zedek Medical Center (SZMC) and Bikur Holim Medical Center (BHMC)—between the years 2005 to 2024. Women were eligible for inclusion if they presented with a twin pregnancy between 24 + 0 and 40 + 6 weeks of gestation, with both fetuses delivered alive (either vaginally or via cesarean delivery), dichorionic or monochorionic diamniotic.

Exclusion criteria included: Maternal anemia due to causes other than iron deficiency, Monoamniotic twins, early fetal reduction from a higher order multiple pregnancy, lethal fetal anomalies or genetic abnormalities of one or both twins and missing maternal data. The study group comprised women diagnosed with anemia upon admission for labor (first lab at admission), defined as hemoglobin levels < 11 g/dL. Additional analyses were performed to the degree of severity. We defined mild anemia (hemoglobin levels 10–11 g/dL), moderate anemia (hemoglobin levels 9–10 g/dL) and severe anemia (hemoglobin levels < 9 g/dL). The control group included women with normal hemoglobin levels at admission.

### Ethical considerations

The study was conducted in accordance with the principles of the Declaration of Helsinki and received approval from our institutional review boards.

### Study outcomes

#### Primary outcome

The primary outcome was a composite adverse neonatal outcome, defined as the presence of at least one of the following:

- 5-minApgar score < 7
- Birth asphyxia
- NICU admission
- Transient tachypnea of the newborn (TTN)
- Mechanical ventilation
- Neonatal encephalopathy
- Intracranial hemorrhage

#### Secondary outcomes:

## Maternal outcomes:

- Gestational age at delivery
- Preterm delivery (PTD)
- Prolonged maternal hospitalization
- Postpartum hemorrhage (PPH)
- Blood product transfusion
- Chorioamnionitis
- Puerperal fever
- Cesarean delivery (overall, intrapartum, and for the second twin)
- Maternal ICU admission

## Neonatal outcomes:

- Birthweight
- Large for gestational age (LGA)
- Small for gestational age (SGA)
- 5-min Apgar score < 7
- NICU admission
- Meconium aspiration syndrome
- Transient TTN
- Mechanical ventilation
- Neonatal encephalopathy
- Intracranial hemorrhage
- Birth asphyxia

### Statistical analysis

Statistical analyses were conducted using SPSS software (version 25; IBM Corp., Armonk, NY, USA). Descriptive statistics were used to summarize categorical and continuous variables. For categorical variables, chi-square or Fisher’s exact tests were applied, as appropriate. For continuous variables, unpaired Student’s *t* test or Mann–Whitney *U* test was used based on data distribution.

Univariate analyses were used to evaluate associations between maternal anemia and demographic, obstetric, and delivery characteristics, comparing the anemic group with the non-anemic control group. All statistical tests were two-sided, and a *p* value of < 0.05 was considered statistically significant.

Variables found to be statistically significant in univariate analyses of the primary and secondary outcomes were included in multivariable logistic regression models. Adjusted odds ratios (aOR) with 95% confidence intervals (CIs) were calculated to assess the independent association between maternal anemia and adverse outcomes.

## Results

**Fig. 1:**
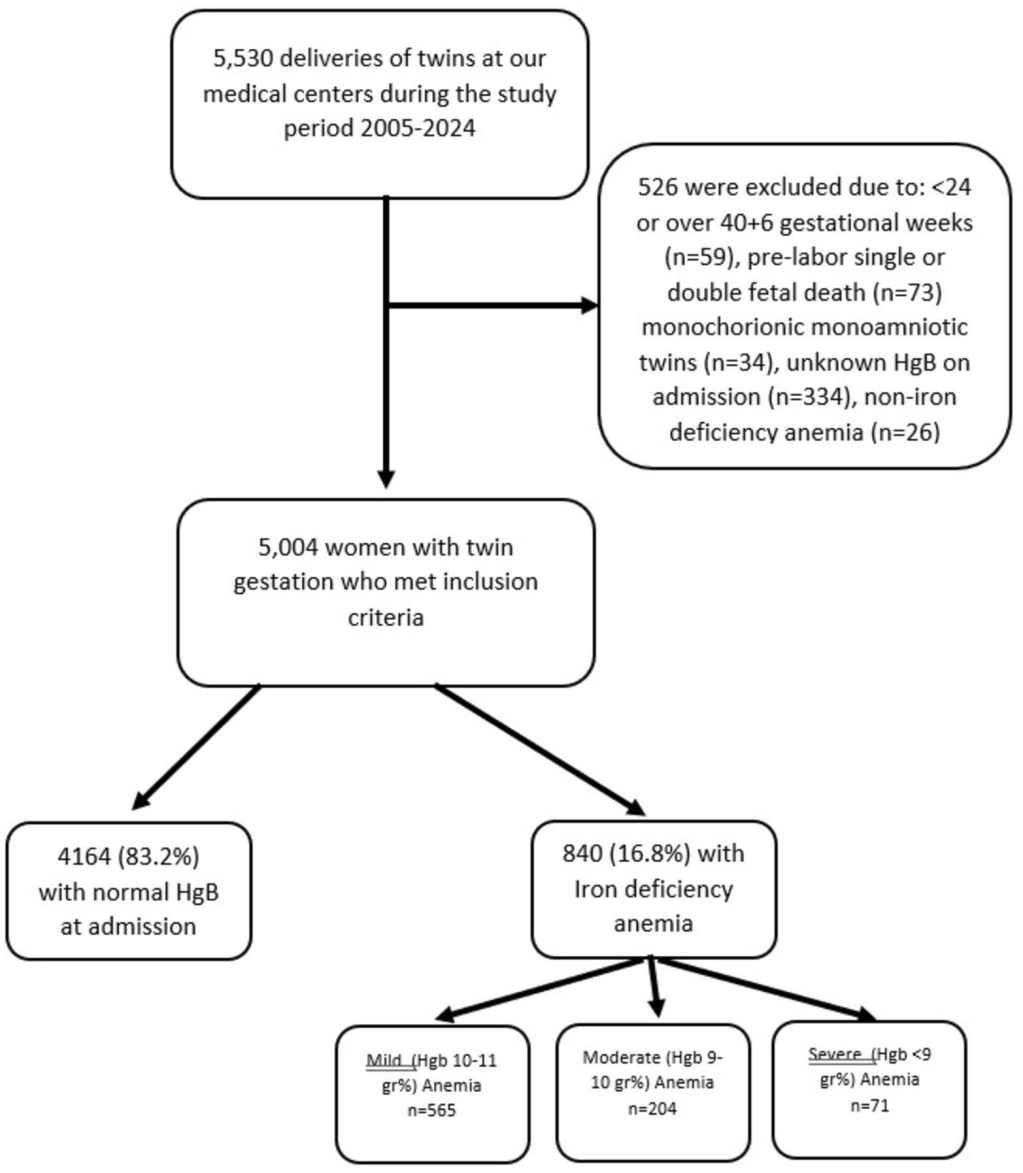
Study population schematic flowchart

During the study period, there were 5530 twin deliveries at the centers included in the study. Of these, 5004 women met the inclusion criteria. The Anemia rate upon admission to labor was 16.8% (*n* = 840), Fig. 1.

### Maternal, pregnancy and delivery characteristics:

Maternal and pregnancy characteristics of women with twin pregnancies, stratified by anemia status, are presented in Table 1. Women with anemia had significantly higher gravidity (4.2 ± 2.8 vs. 3.8 ± 2.8, p < 0.01) and parity (3.5 ± 2.4 vs. 3.3 ± 2.3, p = 0.01) compared to those without anemia. The prevalence of previous CD was higher among women with anemia (17.5% vs. 13.3%, p < 0.01, (3.5% vs. 2.2%, p = 0.05, respectively). No significant differences were observed regarding maternal age, fertility treatments, hypertensive disorders, diabetes disorders, obesity, or induction of labor.

**Table 1:** Maternal and pregnancy characteristics among women with twin pregnancies with and without anemia

	No Anemia * n* = 4164	Anemia * n* = 840	*p* value
Maternal age, years	30.7 ± 5.8	30.8 ± 5.8	0.68
Gravidity	3 [2–5]	3 [2–6]	< 0.01
Parity	3 [1–4]	3 [2–5]	0.01
Primipara	1115 (26.8%)	179 (21.3%)	< 0.01
Previous cesarean delivery, any	553 (13.3%)	147 (17.5%)	< 0.01
Fertility Treatments	1226 (29.5%)	235 (28%)	0.41
Hypertensive disorders of pregnancy	286 (6.9%)	67 (8%)	0.25
Diabetes disorders of pregnancy	371 (8.9%)	61 (7.3%)	0.12
Obesity (BMI > 30)	493 (43.6%)	119 (49.6%)	0.09
Induction of labor	359 (10.2%)	69 (9.7%)	0.73
Hemoglobin at admission	12.5 ± 0.9	10.1 ± 0.8	< 0.01
Monochorionic–diamniotic twins	554 (13.4%)	106 (12.7%)	0.59

Data are mean ± standard deviation, number (%); BMI—Body Mass Index

### Neonatal outcomes:

Neonatal outcomes stratified by maternal anemia status are displayed in Table 2. First and second twins of anemic mothers had significantly lower birth weights (p < 0.01), but with similar rates of small-for-gestational age (SGA) neonates. Anemic mothers' neonates had higher rates of 5-min Apgar scores < 7, jaundice, transient tachypnea of the newborn (TTN), mechanical ventilation, and intracranial hemorrhage (all p < 0.01). In addition, neonatal intensive care unit (NICU) admission rates were higher for both twins (Twin A: 43% vs. 30.2%, p < 0.01; Twin B: 45.4% vs. 33.3%, p < 0.01). The composite adverse neonatal outcome was significantly more frequent in the anemia group for both twins (Twin A: 43.6% vs. 30.9%, p < 0.01; Twin B: 46.9% vs. 34.6%, p < 0.01).

**Table 2:** Comparison of neonatal outcomes among women with twin pregnancies with and without anemia

	No Anemia * n* = 4164	Anemia * n* = 840	*p* value
First twin			
Birthweight, grams	2484.4 ± 484	2345.9 ± 575.1	< 0.01
LGA	516 (12.4%)	123 (14.6%)	0.07
SGA	169 (4.1%)	34 (4%)	0.99
5-Min Apgar score < 7	119 (2.9%)	46 (5.5%)	< 0.01
NICU admission	1257 (30.2%)	361 (43%)	< 0.01
Meconium aspiration syndrome	3 (0.1%)	0 (0%)	0.44
Jaundice	384 (10.2%)	118 (16.1%)	< 0.01
TTN	296 (7.9%)	86 (11.7%)	< 0.01
Mechanical ventilation	245 (6.5%)	104 (14.2%)	< 0.01
Encephalopathy	7 (0.2%)	2 (0.3%)	0.63
Intracranial hemorrhage	45 (1.2%)	36 (4.9%)	< 0.01
Birth asphyxia	105 (2.8%)	29 (4%)	0.09
Composite adverse neonatal outcome*	1285 (30.9%)	366 (43.6%)	< 0.01
Second twin			
Birthweight, grams	2458.3 ± 489.7	2303.8 ± 586.4	< 0.01
LGA	531 (12.8%)	100 (11.9%)	0.50
SGA	272 (6.5%)	54 (6.4%)	0.91
5-Min Apgar score < 7	157 (3.8%)	64 (7.6%)	< 0.01
NICU admission	1384 (33.3%)	381 (45.4%)	< 0.01
Jaundice	399 (10.6%)	134 (18.3%)	< 0.01
TTN	404 (10.7%)	121 (16.5%)	< 0.01
Mechanical ventilation	331 (8.8%)	130 (17.7%)	< 0.01
Encephalopathy	6 (0.2%)	4 (0.5%)	0.04
Intracranial hemorrhage	62 (1.6%)	30 (4.1%)	< 0.01
Birth asphyxia	132 (3.5%)	40 (5.5%)	0.01
Composite adverse neonatal outcome*	1439 (34.6%)	394 (46.9%)	< 0.01

Data are mean ± standard deviation, number (%); *LGA* large for gestational age, *SGA* small for gestational age, *NICU* neonatal intensive-care unit, *TTN* transient tachypnea of the newborn

*Composite adverse neonatal outcome—including at least one of the following: 5-min Apgar score < 7, neonatal asphyxia, NICU admission, transient TTN, mechanical ventilation, encephalopathy, and intracranial hemorrhage

### Maternal and delivery outcomes

Table 3 summarizes the delivery and maternal outcomes. Women with anemia had significantly lower gestational age at delivery (35.3 ± 3.0 vs. 36.4 ± 2.3 weeks, p < 0.01) and increased rates of PTD (< 37 weeks (59.5% vs. 42.5%, p < 0.01), with significantly greater proportion of very PTD (< 34, < 32, and < 28 weeks, all p < 0.01). Anemia was associated with a lower hemoglobin drop after delivery (1.5 ± 1.2 g/dL vs. 1.8 ± 1.4 g/dL, p < 0.01), yet anemic women required more blood transfusions (10.8% vs. 3.1%, p < 0.01). In addition, placental abruption was more frequent in the anemia group (5.7% vs. 2.7%, p < 0.01), and the overall CD rate was higher (59.8% vs. 50.9%, p < 0.01). However, the rate of intra-partum CD was similar between the groups.

**Table 3:** Comparison of delivery and maternal outcomes among women with twin pregnancies with and without anemia

	No Anemia * n* = 4164	Anemia * n* = 840	*p* value
Gestational age at delivery	36.4 ± 2.3	35.3 ± 3	< 0.01
Gestational age at delivery < 37 weeks	1770 (42.5%)	500 (59.5%)	< 0.01
Gestational age at delivery < 34 weeks	389 (9.3%)	162 (19.3%)	< 0.01
Gestational age at delivery < 32 weeks	153 (3.7%)	96 (11.4%)	< 0.01
Gestational age at delivery < 28 weeks	39 (0.9%)	29 (3.5%)	< 0.01
Prolonged hospital stays	428 (10.3%)	98 (11.7%)	0.22
Retained placenta/placental fragments	182 (7.1%)	36 (7.4%)	0.81
Maternal ICU admissions	6 (0.1%)	2 (0.2%)	0.53
Postpartum hemorrhage	608 (14.6%)	97 (11.5%)	0.02
Placental abruption	111 (2.7%)	48 (5.7%)	< 0.01
Hemoglobin drop, gram/dl	1.8 ± 1.4	1.5 ± 1.2	< 0.01
Hemoglobin drop > 3 g/dl	608 (14.6%)	87 (10.4%)	< 0.01
Hemoglobin drop > 4 g/dl	320 (7.7%)	24 (2.9%)	< 0.01
Hemoglobin drop > 5 g/dl	130 (3.1%)	9 (1.1%)	< 0.01
Chorioamnionitis	55 (1.3%)	19 (2.3%)	0.04
Puerperal fever	99 (2.4%)	23 (2.7%)	0.54
Blood products transfusion	131 (3.1%)	91 (10.8%)	< 0.01
Hysterectomy	2 (0%)	1 (0.1%)	0.44
Caesarean, total	2118 (50.9%)	502 (59.8%)	< 0.01
Intrapartum cesarean	157 (7.2%)	15 (4.4%)	0.05
Second twin cesarean	24 (0.6%)	3 (0.4%)	0.43
Data are mean ± standard deviation, number (%); ICU—Intensive Care Unit			

### Impact of anemia severity

Table 4 presents maternal and neonatal outcomes based on anemia severity. Increasing anemia severity was associated with neonatal morbidity, with significantly higher NICU admissions and composite adverse neonatal outcomes (p < 0.01 for all comparisons), as well as with lower gestational age at delivery and PTD (p < 0.01). Severe anemia (< 9 g/dL) was associated with higher rates of placental abruption (9.9%, p < 0.01), blood transfusions (32.4%, p < 0.01), and puerperal fever (7%, p = 0.01).

**Table 4:** Comparison of maternal neonatal outcomes among women with twin pregnancies according to the level of anemia

	No Anemia * n* = 4164	Mild (Hgb 10–11 gr%) Anemia *n* = 565	*p* value	Moderate (Hgb 9–10 gr%) Anemia *n* = 204	*p* value	Severe (Hgb < 9 gr%) Anemia * n* = 71	*p* value
Gestational age at delivery	36.4 ± 2.3	35.5 ± 2.9	< 0.01	34.9 ± 3.1	< 0.01	35 ± 3	< 0.01
Gestational age at delivery < 37 weeks	1770 (42.5%)	326 (57.7%)	< 0.01	130 (63.7%)	< 0.01	44 (62%)	< 0.01
Gestational age at delivery < 34 weeks	389 (9.3%)	100 (17.7%)	< 0.01	44 (21.6%)	< 0.01	18 (25.4%)	< 0.01
Gestational age at delivery < 32 weeks	153 (3.7%)	56 (9.9%)	< 0.01	30 (14.7%)	< 0.01	10 (14.1%)	< 0.01
Gestational age at delivery < 28 weeks	39 (0.9%)	19 (3.4%)	< 0.01	8 (3.9%)	< 0.01	2 (2.8%)	0.11
Prolonged hospital stays	428 (10.3%)	67 (11.9%)	0.24	21 (10.3%)	0.98	10 (14.1%)	0.30
Retained placenta/placental fragments	182 (7.1%)	26 (7.8%)	0.61	10 (8.1%)	0.67	0 (0%)	0.12
Maternal ICU admissions	6 (0.1%)	1 (0.2%)	0.85	1 (0.5%)	0.23	0 (0%)	0.75
Postpartum hemorrhage	608 (14.6%)	63 (11.2%)	0.03	25 (12.3%)	0.35	9 (12.7%)	0.65
Placental abruption	111 (2.7%)	31 (5.5%)	< 0.01	10 (4.9%)	0.06	7 (9.9%)	< 0.01
Hemoglobin drop, gram/dl	1.8 ± 1.4	1.5 ± 1.1	< 0.01	1.5 ± 1.3	0.02	1.7 ± 1.5	0.69
Hemoglobin drop > 3 g/dl	608 (14.6%)	62 (11%)	0.02	17 (8.3%)	0.01	8 (11.3%)	0.43
Hemoglobin drop > 4 g/dl	320 (7.7%)	16 (2.8%)	< 0.01	4 (2%)	< 0.01	4 (5.6%)	0.52
Hemoglobin drop > 5 g/dl	130 (3.1%)	3 (0.5%)	< 0.01	4 (2%)	0.35	2 (2.8%)	0.88
Chorioamnionitis	55 (1.3%)	10 (1.8%)	0.39	7 (3.4%)	0.01	2 (2.8%)	0.28
Puerperal fever	99 (2.4%)	13 (2.3%)	0.91	5 (2.5%)	0.95	5 (7%)	0.01
Blood products transfusion	131 (3.1%)	40 (7.1%)	< 0.01	28 (13.7%)	< 0.01	23 (32.4%)	< 0.01
Hysterectomy	2 (0%)	0 (0%)	0.60	1 (0.5%)	0.02	0 (0%)	0.85
Cesarean, total	2118 (50.9%)	331 (58.6%)	< 0.01	121 (59.3%)	0.02	50 (70.4%)	< 0.01
Intrapartum cesarean	157 (7.2%)	11 (4.6%)	0.13	4 (4.8%)	0.41	0 (0%)	0.21
Second twin cesarean	24 (0.6%)	3 (0.5%)	0.89	0 (0%)	0.28	0 (0%)	0.52
NICU admission twin A	1257 (30.2%)	220 (38.9%)	< 0.01	105 (51.5%)	< 0.01	36 (50.7%)	< 0.01
NICU admission twin B	1384 (33.3%)	236 (41.8%)	< 0.01	107 (52.5%)	< 0.01	38 (53.5%)	< 0.01
Composite adverse neonatal outcome—twin A*	1285 (30.9%)	222 (39.3%)	< 0.01	107 (52.5%)	< 0.01	37 (52.1%)	< 0.01
Composite adverse neonatal outcome—twin B*	1439 (34.6%)	244 (43.2%)	< 0.01	109 (53.4%)	< 0.01	41 (57.7%)	< 0.01

Data are mean ± standard deviation, number (%); ICU—intensive-care unit, NICU—neonatal intensive-care unit

^*^ Composite adverse neonatal outcome—including at least one of the following: 5-min Apgar score < 7, neonatal asphyxia, NICU admission, transient TTN, mechanical ventilation, encephalopathy, and intracranial hemorrhage

### 5. Multivariate analysis

Table 5 presents multivariate logistic regression results for the association between maternal anemia at admission for labor and adverse outcomes. Anemia was independently associated with PTD (< 37 weeks: adjusted OR 2.09, 95% CI 1.79–2.44; < 34 weeks: adjusted OR 2.42, 95% CI 1.98–2.97; < 32 weeks: adjusted OR 3.5, 95% CI 2.67–4.58; < 28 weeks: adjusted OR 3.98, 95% CI 2.44–6.5). Anemia increased the risk of NICU admission (Twin A: adjusted OR 1.84, 95% CI 1.58–2.15; Twin B: adjusted OR 1.77, 95% CI 1.51–2.06) and composite adverse neonatal outcomes for both twins (adjusted OR 1.81 (1.55–2.12) for twin A and adjusted OR 1.77 (1.51–2.06) for twin B) [all p < 0.01]. Anemia was also associated with increased odds of CD (adjusted OR 1.43, 95% CI 1.22–1.68) and blood transfusions (adjusted OR 4.06, 95% CI 3.06–5.38).

**Table 5:** Multivariate logistic regression analyses for the association between maternal anemia at admission for labor and delivery and various maternal and neonatal adverse outcomes among women with twin pregnancies (adjusted odds ratio)

	Adjusted odds ratio (95% confidence interval)
Gestational age at delivery < 37 weeks	2.09 (1.79–2.44)
Gestational age at delivery < 34 weeks	2.42 (1.98–2.97)
Gestational age at delivery < 32 weeks	3.5 (2.67–4.58)
Gestational age at delivery < 28 weeks	3.98 (2.44–6.5)
Postpartum hemorrhage	0.8 (0.63–1.00)
Blood products transfusion	4.06 (3.06–5.38)
Cesarean, total	1.43 (1.22–1.68)
NICU admission twin A	1.84 (1.58–2.15)
Composite adverse neonatal outcome—twin A*	1.81 (1.55–2.12)
NICU admission twin B	1.77 (1.51–2.06)
Composite adverse neonatal outcome—twin B*	1.75 (1.5–2.04)

Adjusted for Gravidity, parity, chronicity and previous cesarean delivery

NICU—neonatal intensive-care unit

^*^ Composite adverse neonatal outcome—including at least one of the following: 5-min Apgar score < 7, neonatal asphyxia, NICU admission, transient TTN, mechanical ventilation, encephalopathy, and intracranial hemorrhage

Table 6 shows a supplementary table that presents multivariate logistic regression results for the association between maternal anemia at admission for labor and adverse outcomes, including adjustment for gestational age. Anemia was independently associated with PTD at all gestational ages as described in Table 5. After adjustment to gestational age anemia was not independently associated with composite adverse neonatal outcomes or NICU admission for both twins, postpartum hemorrhage or cesarean delivery. Anemia was only independently associated with Blood products transfusion (OR 4,11, 95% CI 3.08–5.49).

**Table 6:** Supplementary: Multivariate logistic regression analyses for the association between maternal anemia at admission for labor and delivery and various maternal and neonatal adverse outcomes among women with twin pregnancies (adjusted odds ratio), including gestational age

	Adjusted odds ratio (95% confidence interval)
Association between maternal anemia and preterm delivery*	
Gestational age at delivery < 37 weeks	2.09 (1.79–2.44)
Gestational age at delivery < 34 weeks	2.42 (1.98–2.97)
Gestational age at delivery < 32 weeks	3.5 (2.67–4.58)
Gestational age at delivery < 28 weeks	3.98 (2.44–6.5)
Association between maternal anemia and various maternal and neonatal adverse outcomes**	
Postpartum hemorrhage	0.82 (0.65–1.04)
Blood products transfusion	4.11 (3.08–5.49)
Cesarean, total	1.14 (0.97–1.34)
NICU admission twin A	0.91 (0.7–1.18)
Composite adverse neonatal outcome—twin A***	0.9 (0.7–1.15)
NICU admission twin B	0.9 (0.71–1.13)
Composite adverse neonatal outcome—twin B***	0.93 (0.74–1.16)

^*^Adjusted for Gravidity, parity, chronicity and previous cesarean delivery

^**^Adjusted for Gravidity, parity, chronicity, previous cesarean delivery and gestational age at delivery

NICU Neonatal intensive-care unit

^*^ Composite adverse neonatal outcome—including at least one of the following: 5-min Apgar score < 7, neonatal asphyxia, NICU admission, transient TTN, mechanical ventilation, encephalopathy, and intracranial hemorrhage

## Discussion

### Principal findings

In this multicenter retrospective cohort study, maternal anemia in twin pregnancies (defined based on hemoglobin levels upon admission for labor), was associated with increased rates of adverse maternal and neonatal outcomes. Women with anemia had higher rates of preterm delivery (PTD), placental abruption, cesarean delivery (CD), and maternal blood transfusions compared to women with normal hemoglobin levels. Neonates born to anemic mothers—both twins—were more likely to experience composite adverse neonatal outcomes, including 5-min Apgar scores < 7, jaundice, transient tachypnea of the newborn (TTN), mechanical ventilation, intracranial hemorrhage, and NICU admission. Furthermore, increasing anemia severity was correlated with a progressive rise in these adverse outcomes. Multivariable analysis confirmed that anemia was independently associated with composite adverse neonatal outcomes, NICU admission, PTD, CD, and maternal blood transfusion.

### Results in the context of what is known:

Previous research has established that maternal anemia in singleton pregnancies is associated with elevated risks of PTD, placental abruption, hypertensive disorders, postpartum hemorrhage, low birthweight, stillbirth, and maternal mortality[2, 3, 5, 6]. Although anemia is more prevalent in twin pregnancies than in singleton,[19, 20] its specific impact in twin gestations has been underexplored. Given that twin pregnancies inherently carry higher baseline risks for complications, such as PTD, pre-eclampsia, and postpartum hemorrhage,[17] examining the intersection of these conditions has significant clinical relevance [21–25].

However, the existing literature is limited due to small sample sizes and inconsistent findings. Lin et al. demonstrated that anemia in twin pregnancies was associated with low 1-min Apgar scores, lower birthweights, NICU admission, and perinatal death, and that correction of anemia in the third trimester improved outcomes [21]. Yet, after adjustment for confounders, only NICU admission remained statistically significant. Similarly, Samita et al. reported that maternal anemia was the most common antepartum complication that contributed to PTD in multiple pregnancies [25].

Conversely, Kosto et al. found no significant association between anemia in twin gestation and most maternal or neonatal outcomes, except for increased blood transfusion risk; however, their study included only 66 women with anemia [22] Mathew et al. and Kristi et al. also failed to find significant associations with PTD or adverse neonatal outcomes [23, 24], and Kristi et al. even suggested a paradoxical association between hemoglobin drop and higher gestational age. These inconsistencies are likely due to limited sample sizes and lack of stratification by anemia severity. This study also showed a paradoxical correlation with lower rates of hemoglobin drop, but higher rates of blood product transfusion in anemic women. It is possible that the decrease in hemoglobin levels should be counted in percentages from each women base, and not necessarily in a numerical delta, which might reflect more reliably postpartum hemorrhage and predict need of blood product transfusion. However, further studies are needed to understand this association.

The major innovation of this study lies in its large cohort—comprising over 5,000 twin pregnancies, including 840 cases with anemia—which providing the statistical power to detect associations across a spectrum of maternal and neonatal outcomes. Our findings also align with biological plausibility: It has already been shown in several studies that anemia in singleton pregnancy increases the risk of PTD [2], and that also twin gestation is associated with increased PTD risk [17, 26, 27]. Therefore, it is reasonable that the combination of these two conditions—anemia and twin pregnancy—will increase the risk. Since PTD is a well-established risk factor for neonatal morbidity [28], the higher PTD rates in this study may partly explain the elevated risk of adverse neonatal outcomes.

Therefore, we also performed another multivariate analysis that included adjustment for gestational age. After adjustment for gestational age, anemia in twin gestation was not found to be independently associated with neonatal complications for both twins, or with maternal complications except for increased blood transfusion requirements. It seems that indeed—most of the association between anemia and adverse neonatal outcomes is contributed from the increased risk of preterm delivery, with its inherent risks [28–30]. However, clinical significance remains the same despite this clarification, as there is still higher risk for adverse neonatal and maternal outcomes in twin pregnancies complicated with anemia, and close monitoring and appropriate management are needed.

We also found that maternal anemia was independently associated with higher rates of overall CD. A similar finding was reported in singleton pregnancies with anemia [7] This correlation was not demonstrated in intrapartum CD. The underlying mechanism of this association remains unclear but may reflect residual confounding or unmeasured clinical factors. It might also suggest provider and patient preference for a controlled delivery setting in the presence of maternal anemia, particularly in already high-risk twin gestations. This association warrants further investigation.

### Clinical implications

Our findings underscore the importance of recognizing and addressing anemia in twin pregnancies. The observed associations with PTD, CD, maternal transfusion, and NICU admission suggest that women with anemia require closer monitoring during pregnancy, labor, and the postpartum period. Proactive identification and correction of anemia may help mitigate these risks.

### Research implications

This study contributes new insights by focusing on the understudied subgroup of twin pregnancies complicated by maternal anemia. Future research should aim to validate these findings in other populations and explore the pathophysiological mechanisms underlying these associations. Specifically, the timing and etiology of anemia, as well as the impact of anemia treatment, should be investigated in prospective studies to inform targeted interventions.

### Strengths and limitations

A key strength of this study is the large sample size, which enabled stratification by anemia severity and robust multivariable analysis across a wide range of outcomes. The use of comprehensive, real-time electronic medical records enhanced data accuracy and completeness.

However, the retrospective design is a limitation, with inherent risks of confounding and missing data. Another limitation of this study is the reliance on hemoglobin levels measured upon admission for labor, which may not accurately reflect chronic or earlier third-trimester anemia status. This study lacks data regarding the duration or progression of anemia during pregnancy. Therefore, our findings primarily reflect the association between anemia upon admission for labor and adverse outcomes and may not capture the full impact of antenatal anemia. Another limitation of this study is the lack of data regarding the etiology of anemia, or treatments during pregnancy, including iron supplementation or other therapeutic interventions. Without this data, we could not assess the impact of treatment on maternal or neonatal outcomes. Prospective studies are needed to explore these variables and their relationship with clinical outcomes.

## Conclusion

In this multicenter retrospective cohort study, maternal anemia in twin pregnancies was associated with increased risks of composite adverse neonatal outcomes, NICU admission, PTD, CD, and maternal blood transfusions. These findings may indicate a possible clinical relevance for identifying and treating anemia in twin pregnancies. Early intervention and tailored monitoring, when appropriate, could potentially contribute to improved maternal and neonatal outcomes.

## Data Availability

Data is anonymized and is available at the request of the reviewers.
